# Recent advances in membrane mimetics for membrane protein research

**DOI:** 10.1042/BST20230164

**Published:** 2023-06-22

**Authors:** John William Young

**Affiliations:** Department of Chemistry, Kavli Institute for Nanoscience Discovery, Dorothy Crowfoot Hodgkin Building, University of Oxford, South Parks Rd, Oxford OX1 3QU, U.K.

**Keywords:** membrane mimetics, membrane protein reconstitution, membrane proteins, nanodiscs, peptidiscs

## Abstract

Membrane proteins are a highly relevant class of biological molecules and comprise ∼60% of current drug targets. Before being analyzed by structural, biochemical, and biophysical methods, membrane proteins must first be extracted from cellular membranes — often using detergents. Detergent-extracted membrane proteins are amenable to analysis by structural, biochemical, and biophysical techniques. In certain cases, however, detergents can disturb native protein conformations and/or biological activity. This has led to the development of membrane mimetics, which stabilize membrane proteins in a native membrane-like environment that is water-soluble and detergent-free. This review provides an overview of recent developments in the membrane mimetic field, with a focus on nanodiscs, Saposin lipid nanoparticles (SapNPs), peptidiscs, and SMA lipid particles (SMALPs) — and highlights their utility for supporting biophysical, biochemical, and structural characterization of membrane proteins and complexes.

## Introduction

Integral membrane proteins are among the most therapeutically relevant biological molecules and control many of the essential processes of life including energy production, nutrient import, cell–cell signaling, and protein translocation [1–3]. Their importance for maintaining cellular homeostasis is highlighted by the fact that membrane proteins comprise ∼60% of current drug targets [4].

Before being analysed by most structural or biochemical methods, membrane proteins must first be extracted from the hydrophobic lipid bilayer. This is typically done using detergents, which solubilize the lipid bilayer and maintain membrane proteins in a soluble state by shielding their hydrophobic surfaces from water [5,6]. Detergent-solubilized membrane proteins are amenable to structural analysis by methods such as X-ray crystallography and single-particle cryo-electron microscopy (Cryo-EM) [7–12]. They are also amenable to native mass spectrometry (nMS) and receptor–ligand binding assays [13–17]. However, even the mildest detergents can disrupt native protein conformations, or else strip away functionally important phospholipid and/or protein interactors [15,18–21]. Extensive efforts have been directed towards developing new classes of detergents to minimize these dissociating and delipidating effects [22–24]. The reader is referred to the following excellent reviews for more details on advances in detergent chemistry [25,26].

To minimize these detergent effects, or else bypass them altogether, a major priority for the membrane protein research field has been the development of ‘membrane mimetics’ which can stabilize membrane proteins in a native membrane-like environment that is detergent-free and fully water-soluble [3]. This review aims to provide an overview of the latest developments in the membrane mimetic field, particularly focussing on nanodiscs, Saposin lipid nanoparticles (SapNPs), peptidiscs, and SMA lipid particles (SMALPs). Other widely used membrane mimetics, notably including liposomes, are not discussed in detail here due to space considerations but have been extensively described elsewhere [27,28].

## Nanodiscs

**Figure 1.**
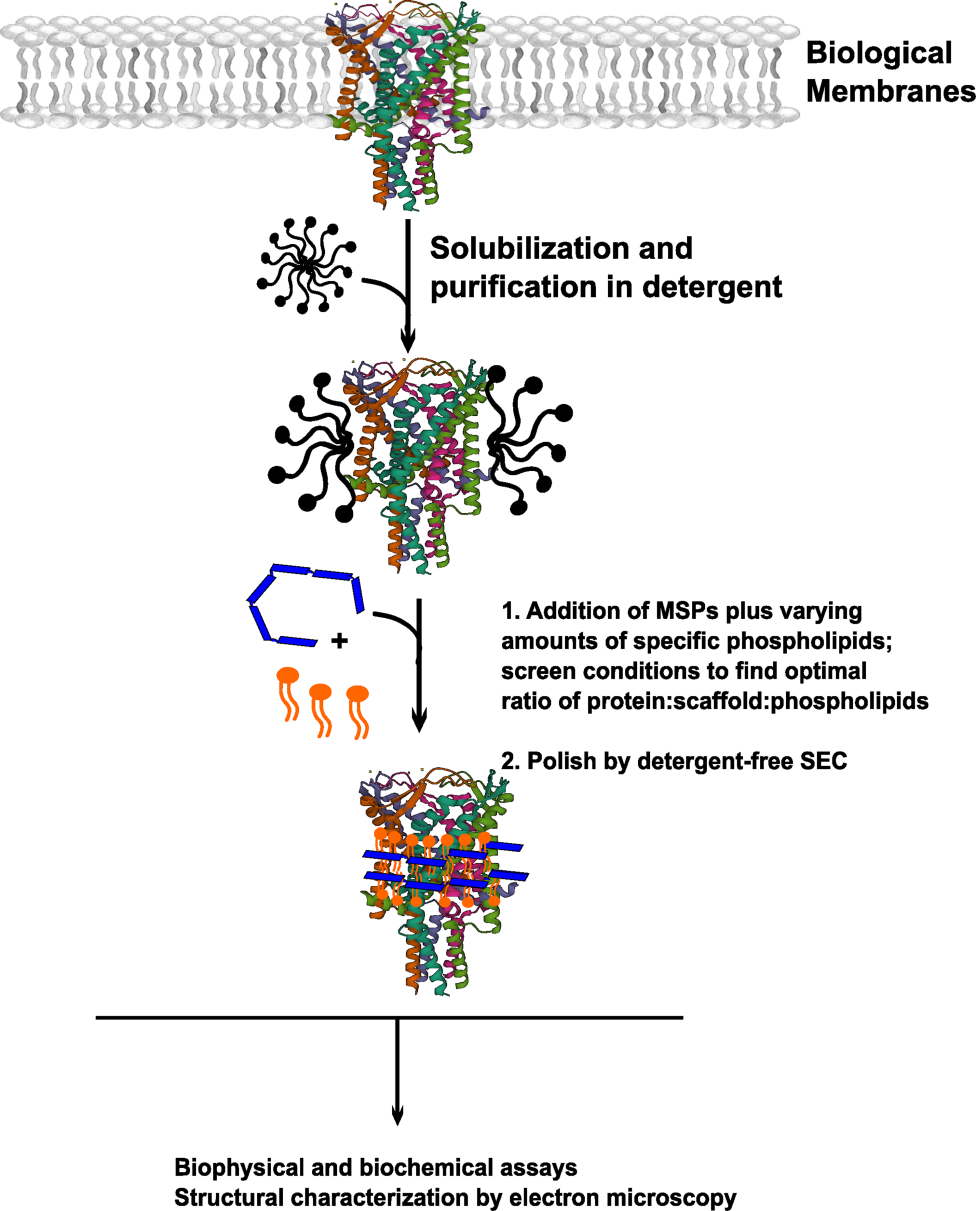
Flowchart depicting the nanodisc reconstitution workflow. Biological membranes containing the membrane protein of interest are solubilized in mild detergent, and the target protein is purified. Multiple steps are often required to maximize purity: most often an affinity purification, followed by SEC. Once the target protein is sufficiently pure, researchers screen for ideal reconstitution conditions — typically trying different lengths of membrane scaffold protein (MSP — depicted in blue), and varying amounts of phospholipids (depicted in orange). Reconstitution quality is assessed by SEC and by negative stain EM. The resulting nanodisc particles are amenable to a wide range of downstream applications, from high-resolution structural characterization by Cryo-EM to antibody discovery.

The most well established and widely used membrane mimetic system covered in this review is the nanodisc, which was first described by the Sligar laboratory in 2002 [29]. Nanodiscs are small, water-soluble patches of lipid bilayer containing a membrane protein of interest encircled by two copies of an ApoA1-derived membrane scaffold protein (MSP) [29,30]. In the last two decades, they have been widely used as a platform to facilitate structural, biochemical, and biophysical analysis of membrane proteins and complexes [31,32]. In the standard workflow for membrane protein reconstitution into nanodiscs, a detergent-purified target protein is incubated with detergent-solubilized phospholipids and MSPs (Figure 1). To promote disc formation, the detergent is gradually removed by dialysis or by incubation with BioBeads, and the nanodisc-reconstituted target protein is further purified by size-exclusion chromatography (SEC) in detergent-free buffer to remove aggregates and separate the reconstituted target protein from ‘empty’ lipid-only discs and/or excess MSPs [33,34]. Several key parameters must be optimized to ensure successful reconstitution of a target protein into homogenous nanodisc particles. First and foremost is the length of the scaffold protein included in the reconstitution mixture. Various MSP constructs — containing multiple ApoA1-derived alpha-helices — have been developed, allowing the user to precisely control the diameter of the nanodisc particle [35]. These MSPs produce nanodiscs with diameters varying between 8 and 16 nm [3,30,35]. More recently, researchers have also introduced covalently circularized MSPs, using either Sortase or SpyCatcher technology to form a covalent bond between the N- and C-termini of an MSP [36,37]. Nanodiscs formed using these circularized scaffolds have greater homogeneity compared with those formed using a ‘conventional’ linear MSP [36,38]. Importantly, the circularized MSP technology enables formation of much larger nanodiscs, with diameters of up to 50 nm [36,37]. This latter feature is potentially critical for reconstituting large multi-subunit membrane protein complexes, which may be too large to be accommodated by a shorter, linear MSP [38]. Conveniently, all these MSP variants can be obtained with high yield and purity after expression in *Escherichia coli* (*E. coli*) [30,35,37]. This feature enables researchers to rapidly screen different reconstitution scaffolds with only minimal cost.

Because phospholipids can strongly influence the activity of the reconstituted target protein, a second parameter requiring optimization is the identity and number of phospholipids to include in the reconstitution mixture. Depending on the research question, researchers may add pure synthetic phospholipids during reconstitution, or else a more complex lipid extract [39–41]. The ratio of target protein to phospholipids and MSPs needed for successful reconstitution into homogenous particles must be carefully optimized and can be difficult to predict — researchers often need to screen several different mixtures by ‘trial and error’ to find the best conditions. This process can be time-intensive and consumes large amounts of valuable membrane protein sample. Reconstitution conditions can be screened using techniques including non-denaturing gel electrophoresis, SEC, light scattering coupled to SEC (SEC-MALS), negative stain electron microscopy (NS-EM), and mass photometry (MP) [42–45]. Once optimal conditions are found, the preparation can be scaled up for downstream structural and/or biochemical analysis.

In recent years nanodiscs have emerged as a robust platform for supporting high-resolution structural characterization of membrane proteins and complexes, particularly by single-particle Cryo-EM. Following recent advances in Cryo-EM imaging technology, which have enabled a ‘resolution revolution’, there has been a dramatic increase in the number of high-resolution structures of nanodisc-embedded membrane proteins and complexes deposited in the PDB [3]. Nanodiscs can be applied to characterize membrane proteins expressed in prokaryotes and eukaryotes; notable recent structures include various transporters, ion channels, and G-protein-coupled receptors (GPCRs) [41,46–48]. Many of these structures have revealed new insights into membrane protein structure and function which were not previously evident from studies performed in detergent micelles — such as revealing the binding sites for specific lipids which modulate protein function or revealing a substrate binding site [48–50].

Beyond high-resolution structural characterization of membrane proteins, nanodiscs have been used to characterize binding of soluble protein and small-molecule ligands onto membrane protein receptors. The water-soluble environment provided by the nanodisc is invaluable for characterizing these binding events in the absence of detergents [33,44,51–54]. ‘Empty’ lipid-only nanodiscs have also been used to monitor binding of peripheral membrane proteins onto membrane lipids [40,55]. This application makes nanodiscs particularly useful in the context of peripheral membrane proteins involved in signaling pathways, as their activity is often influenced by specific phospholipids [56]. In another recent development, the Marty laboratory recently showed that lipid-only nanodiscs can be used together with nMS to study membrane binding of antimicrobial peptides [39]. This latest application may be especially impactful for developing novel antibiotics. One potential caveat to note, however, is that nanodiscs are unable to mimic membrane curvature, nor lipid asymmetry in cellular membranes — both of which can modulate binding of peripheral membrane proteins. Researchers should be aware of this potential shortcoming when using lipid nanodiscs to study binding of soluble effector proteins onto cellular membranes.

Despite its impressive track record in enabling structural and biophysical characterization of membrane proteins, a major shortcoming of the nanodisc workflow is that the membrane protein of interest must be fully purified in detergent — which often requires multiple chromatography steps — prior to nanodisc reconstitution. Since prolonged exposure to detergents can strip away functionally important annular lipids and disrupt labile, multi-subunit protein complexes [18,21], the classical nanodisc workflow is not always suitable for characterizing fragile, detergent-sensitive membrane protein assemblies. In the sections below, I will outline recent developments with alternative membrane mimetic systems which minimize exposure of the target protein or complex to detergents. These alternatives to the classical nanodisc may be more suitable for characterization of detergent-sensitive multi-subunit membrane protein complexes.

## Salipro

**Figure 2.**
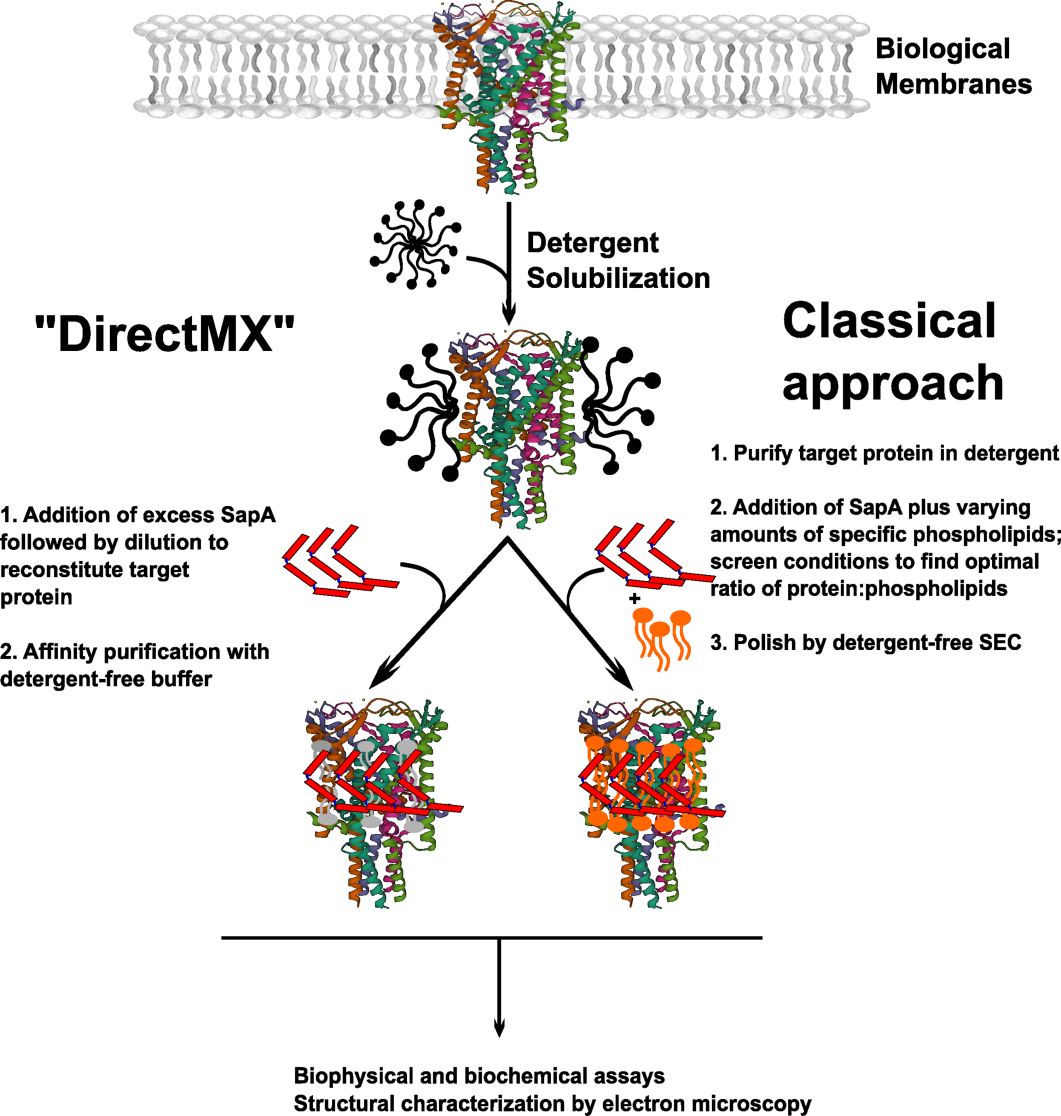
Flowchart depicting two Salipro reconstitution strategies. Biological membranes containing the membrane protein of interest are solubilized in mild detergent. In the most widely used ‘classical’ approach (shown in the right flow-path), the target protein is first purified in detergent, then researchers screen for ideal reconstitution conditions — typically adding an excess of SapA scaffold protein (depicted in red), and varying amounts of phospholipids (depicted in orange). In the recently developed ‘DirectMX’ approach (left flow-path), an excess of SapA is added to the detergent-solubilized membranes, and reconstitution is achieved by dilution with aqueous buffer. The reconstituted protein, along with any tightly bound annular lipids (shown in gray) is then affinity purified in detergent-free conditions.

Introduced in 2016 by Jens Frauenfeld and coworkers, the Salipro membrane mimetic system is based on the mammalian lipid-binding protein Saposin A (SapA), which can be produced in high yield and purity following expression in *E. coli* [57]. Saposin Lipid Nanoparticles — sometimes termed ‘SapNPs’ — consistent of a target membrane protein embedded in a small patch of lipid bilayer surrounded by multiple copies of SapA [57,58]. The standard workflow for membrane protein reconstitution into SapNPs resembles that for an MSP nanodisc (Figure 2): a detergent-purified membrane protein of interest is incubated with a pre-determined ratio of phospholipids and SapA scaffold protein. Following detergent removal by dialysis or incubation with BioBeads, downstream purification by SEC in detergent-free buffer is routinely performed to separate SapNPs containing the protein of interest from ‘empty’ lipid-only SapNPs and excess SapA [57–59].

A distinct advantage of SapNPs over nanodiscs is that SapA is a flexible scaffold and can adapt to the size of the target membrane protein, adjusting to transmembrane regions of varying sizes. This simplifies the reconstitution optimization process by eliminating the need to screen different polypeptide scaffolds of varying lengths [59]. However, the ratio of phospholipids and SapA to target protein in the reconstitution mixture of interest must still be carefully tuned to ensure formation of homogenous SapNPs [58]. As with nanodiscs, the quality of membrane protein reconstitution into SapNPs can be verified using methods including SEC, light scattering coupled to SEC (SEC-MALS), mass photometry (MP), and NS-EM [57,59,60].

SapNPs are amenable to high-resolution structural characterization by single-particle Cryo-EM. Notable examples of membrane proteins whose structures were determined using the Salipro system include the bacterial membrane proteins MsbA, AcrB, and BamABCDE [61–63]; and the eukaryotic 5HT3A serotonin receptor, the ion channel ASOR, and the nicotinic acetylcholine receptor [64–66].

Owing to the flexibility of the SapA scaffold, the Salipro system represents an attractive alternative to the classical MSP nanodisc. As with nanodiscs; however, a shortcoming of the established SapNP reconstitution workflow is that the membrane protein of interest must be fully purified in detergent prior to reconstitution. Is it possible to reconstitute membrane proteins into SapNPs while avoiding extensive exposure of the target protein to detergents? In an exciting new development, researchers at Salipro Biotech — a company commercializing the Salipro technology — recently introduced a method called ‘DirectMX’, which enables reconstitution of membrane proteins into SapNPs directly from cellular membranes (Figure 2) [67]. For membrane protein reconstitution by DirectMX, membranes containing an affinity-tagged protein of interest are solubilized with a mild detergent, such as digitonin. Reconstitution into SapNPs is achieved by diluting the solubilized material in a large volume excess of aqueous buffer containing pure SapA. Following a brief incubation, the reconstituted target protein is purified by affinity chromatography in detergent-free conditions [60,67].

Very encouragingly, membrane proteins purified by this method appear homogenous when analysed by SEC and NS-EM [67]. In a recent study on the mammalian PANX1 channel, Drulyte et al. [60] further show that SapNPs prepared by DirectMX are amenable to high-resolution structural characterization by Cryo-EM, as well as ligand binding assays by SPR. While DirectMX was introduced very recently and has so far been employed on only a handful of targets, the method appears very promising for stabilization, purification, and downstream characterization of detergent-sensitive membrane protein assemblies. In particular, DirectMX may be useful for purification and reconstitution of human membrane proteins which are directly implicated in health and disease — including GPCRs and ion channels [67]. While highly therapeutically relevant, these proteins can be unstable in detergent solution and are thus difficult to obtain in a water-soluble and biologically active format for downstream small molecule and/or antibody discovery experiments [46,67]. Since the target protein is exposed to detergent for only a short time with DirectMX, this method may be widely applicable for preparing challenging human membrane protein targets in a format that is suitable for downstream drug discovery experiments — including screening for small molecule binders and for therapeutic antibodies. The field of antibody discovery against membrane protein targets is growing rapidly and is a very promising area for development of new therapeutics [68,69].

## Peptidiscs

A second recently developed alternative to the nanodisc method is the peptidisc, which was introduced by the Duong laboratory in 2018 [42]. Peptidisc reconstitution is based on a 37-amino acid amphipathic ApoA1-mimetic peptide termed ‘NSP’ (nanodisc scaffold peptide) [70]. To increase the water-solubility of the peptide scaffold, Carlson et al. reversed the amino acid sequence of NSP, generating the peptide ‘NSP_r_’ (nanodisc scaffold peptide, reversed) — often termed the ‘peptidisc’ peptide [20,42].

Recent work has shown that peptidiscs can be used to reconstitute membrane proteins with varying sizes and topologies from both eukaryotic and bacterial sources and appears to require only minimal optimization for a given target protein [42,71–73]. Membrane proteins reconstituted into peptidiscs are amenable to high-resolution structural analysis by electron microscopy, as well as a variety of biophysical and biochemical analyses — including ATPase activity assays, and receptor–ligand interaction studies using techniques including biolayer interferometry (BLI) [20,71–75].

**Figure 3.**
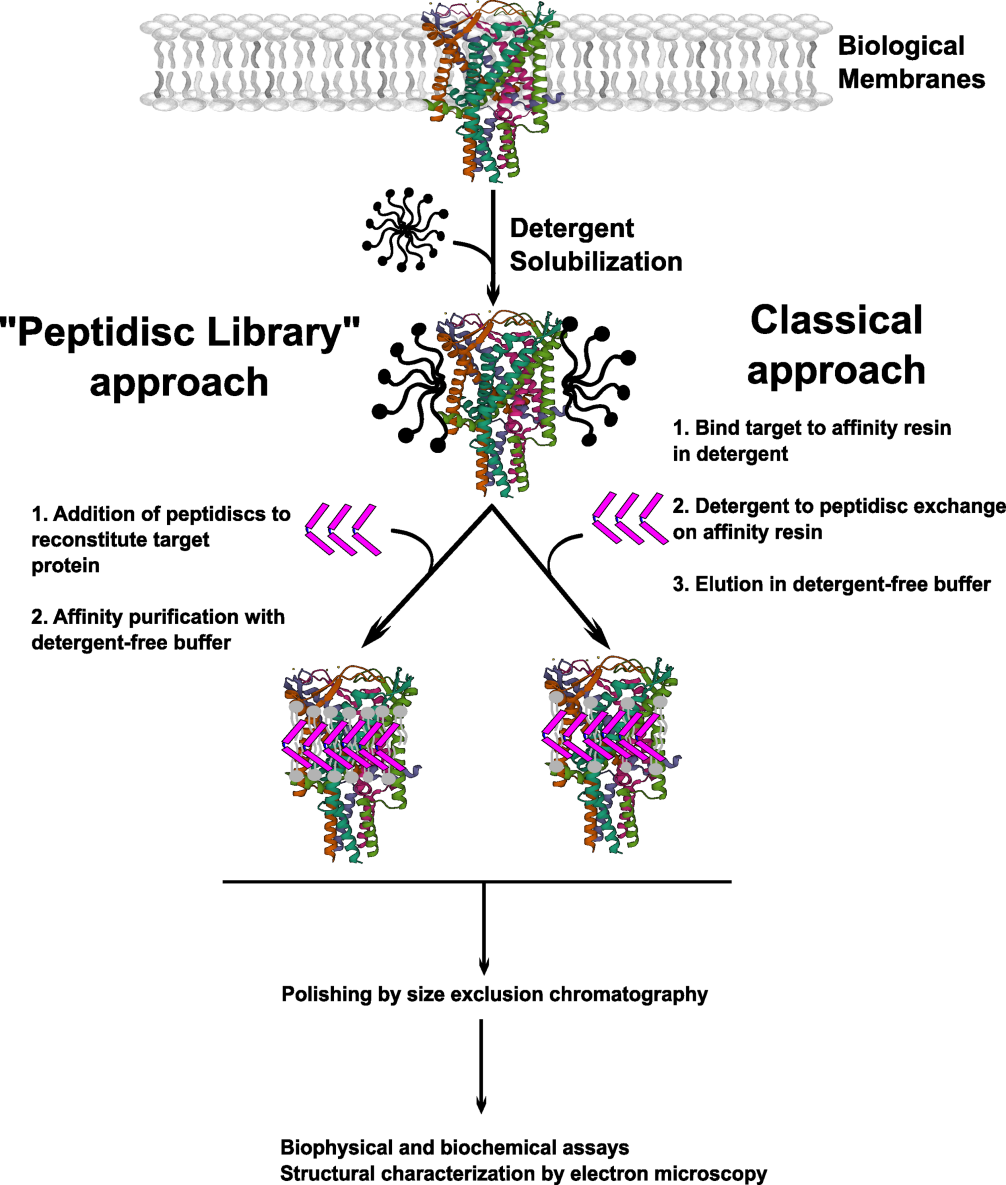
Flowchart depicting two Peptidisc reconstitution strategies. Biological membranes containing the membrane protein of interest are solubilized in mild detergent. In the classical approach (shown in the right flow-path), the target protein is bound to affinity resin in detergent. Peptidiscs are then substituted for detergent during the washing steps. After washing in detergent-free buffer to remove excess peptides, the reconstituted target protein — along with tightly bound annular lipids (shown in gray) is eluted in detergent-free buffer. In the alternative ‘Peptidisc Library’ approach (left flow-path), an excess of Peptidisc peptides (shown in purple) is added to the detergent-solubilized membranes, and reconstitution is achieved by dilution with aqueous buffer. The reconstituted protein, along with any tightly bound annular lipids (shown in gray) is then affinity purified in detergent-free conditions.

The procedure for membrane protein reconstitution into peptidiscs appears more streamlined compared with either nanodiscs or Salipro and does not require addition of specific phospholipids. The peptidisc scaffold itself is highly flexible and adapts to the size of the transmembrane regions of the target protein [20,42]. In a typical experiment, reconstitution is achieved during affinity purification using a protocol termed ‘on-beads’ reconstitution [20,42,74]. Here, the detergent-solubilized target protein is bound to affinity resin, and contaminants are removed by washing with detergent-containing buffer. The resin is then incubated with a large volume excess of detergent-free buffer supplemented with concentrated peptidisc peptide. As the detergent concentration is diluted well below the critical micellar concentration (CMC), multiple copies of the peptidisc peptide can self-assemble around the hydrophobic transmembrane region of the target protein and any co-purifying annular lipids, forming homogenous peptidisc particles (Figure 3). After a brief wash step to remove excess peptidisc peptides, the reconstituted membrane protein is eluted in detergent-free buffer [20,42,74]. The quality of the reconstituted material can then be verified by SEC and NS-EM [20,42].

In this author's experience, the ‘on-beads’ reconstitution approach routinely yields highly homogenous, biologically active peptidisc-encapsulated membrane proteins. In certain cases, however, this strategy can result in extensive aggregation of the protein sample. In these cases, peptidisc reconstitution can be achieved by incubating the detergent-purified membrane protein with an excess of the peptidisc scaffold, then fractionating the mixture in detergent-free conditions by either SEC or density gradient centrifugation [20,42,72].

Like Salipro, recent work has shown that peptidiscs can be used to reconstitute membrane proteins immediately after their extraction from the lipid bilayer with mild detergents [18,76,77]. This feature can be very beneficial for stabilizing and purifying multi-subunit protein complexes which would otherwise dissociate upon prolonged detergent exposure [21]. Biological membranes containing an affinity-tagged protein of interest are solubilized with a mild detergent before addition of an excess of peptidisc peptides (Figure 3). The detergent concentration is immediately diluted below the CMC by addition of aqueous buffer, and the sample is concentrated to remove excess detergent micelles. Multiple rounds of the dilution/concentration steps results in formation of a water-soluble membrane protein library, or a ‘peptidisc library’. Following library formation, the affinity-tagged target protein can then be purified in detergent-free conditions [18,21]. While proteins purified in this manner are water-soluble [18,21,76], no high-resolution structural data has yet been reported for a membrane protein or complex purified from a peptidisc library. It should also be noted that although the ‘peptidisc library’ approach for membrane protein characterization has been extensively applied towards bacterial membrane proteins [21,76,77], it has yet to be applied to eukaryotic membrane proteins.

## SMALPs

**Figure 4.**
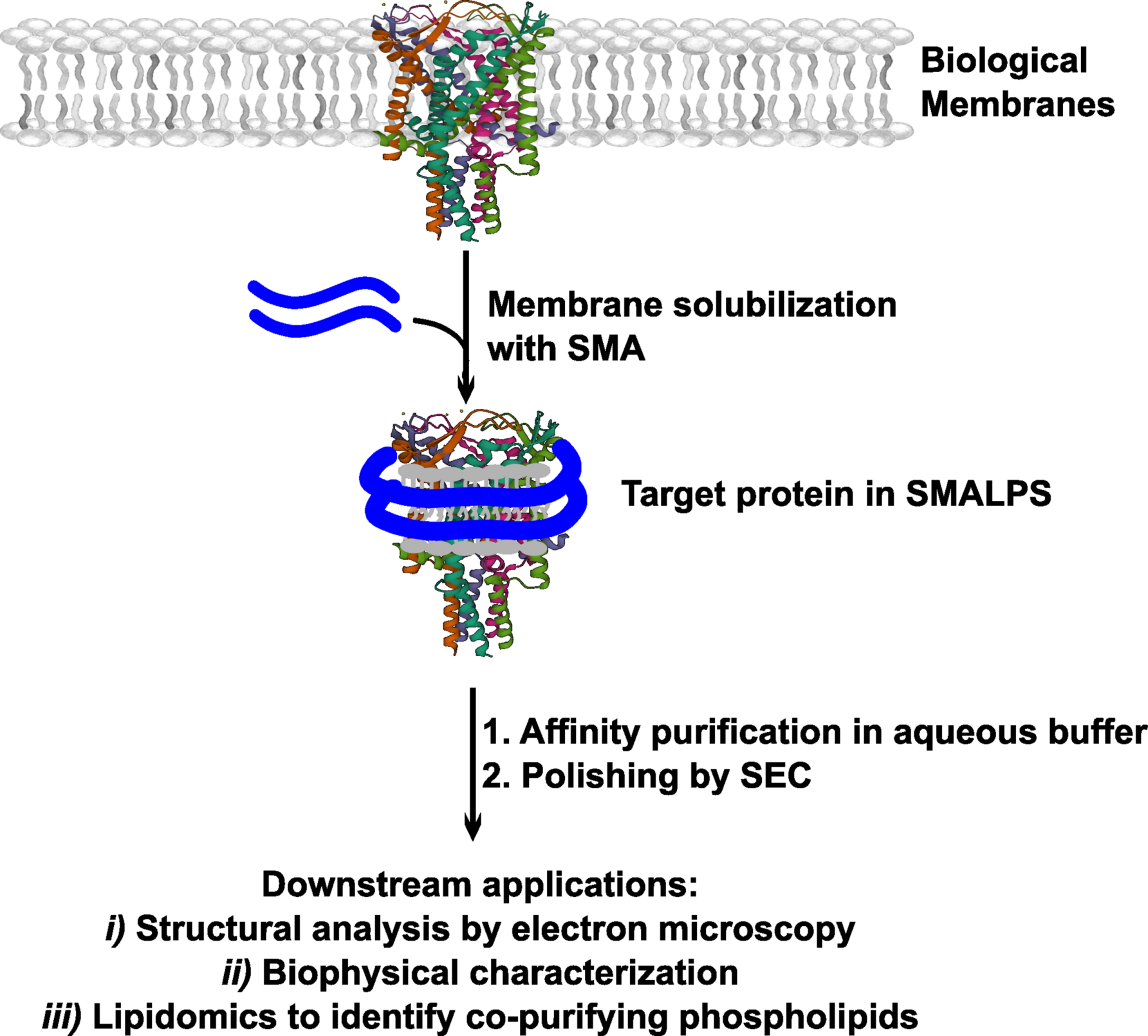
Flowchart depicting the SMALPs reconstitution workflow. Biological membranes containing the membrane protein of interest are solubilized using a SMA polymer, and the target protein is purified in detergent-free conditions. Multiple steps are often required to maximize purity: most often an affinity purification, followed by SEC. Once the target protein is sufficiently pure, it can be used in a variety of downstream applications, some of which are listed here.

SMALPs — often termed ‘Native Nanodiscs’ or ‘Lipodisqs’ — are based on an amphipathic styrene maleic acid (SMA) copolymer [78,79]. SMALPs have attracted considerable interest because the SMA polymer — unlike nanodiscs, saposins, and peptidiscs — can extract membrane proteins directly from the lipid bilayer in the absence of detergents, encapsulating them into discoidal, water-soluble particles (Figure 4) [5,78,80,81]. Thus, the SMALPs system is the only membrane mimetic that is truly ‘detergent-free’. Following the initial extraction step, a tagged protein of interest is affinity purified in detergent-free conditions. A subsequent SEC step is routinely performed to remove aggregates and/or stubborn protein contaminants [78,82]. Interactions between the target protein and its surrounding lipid environment — which are often critical for protein function — are preserved upon extraction with SMA and during the subsequent purification steps [80,82–85].

SMALPs have been successfully applied to purify and characterize integral membrane proteins from both bacterial and eukaryotic systems. SMALP-reconstituted membrane proteins are amenable to high-resolution structural characterization by Cryo-EM, as well as receptor–ligand binding assays and activity assays [82,85–87].

While SMALPs have been successfully used for structural, biophysical, and biochemical analysis of membrane proteins in recent years, it is also important to note some limitations to the technique. The solubilization efficiency of the polymer is often lower than for commonly used detergents [88]. This low solubilization efficiency is especially problematic in the context of eukaryotic membrane proteins, which are often present at low levels [3]. Due to the negative charge on the polymer, SMA can interfere with binding of a tag onto affinity resin, particularly during metal affinity chromatography [84]. An additional notable shortcoming is that the polymer is prone to precipitation in the presence of divalent cations including Mg^2+^ and Ca^2+^ [88]. Since these divalent cations are essential in numerous biochemical assays such as ATPase assays, this drawback can seriously complicate biochemical analysis of purified membrane transporters, such as members of the ABC transporter family [3,6]. While several new polymer derivatives have been developed recently with reduced sensitivity to divalent cations, this represents an ongoing area of development in the SMALPs research field.

## Summary and conclusion

Membrane proteins play critical roles in many cellular processes, and their mis-function or mis-regulation is linked to many debilitating human disorders, including cystic fibrosis, heart disease, and loss of vision [89–91]. However, membrane proteins are challenging to study because they are embedded in cellular membranes. Before being analysed by most structural and biophysical methods membrane proteins must first be extracted — or ‘solubilized’ — from the hydrophobic interior of the membrane, often using detergents. Although highly effective for solubilizing biological membranes, detergents do not faithfully mimic a lipid-rich native membrane environment and can have adverse effects on membrane protein structure and function [15].

To facilitate the study of membrane proteins in the absence of detergents, researchers have developed ‘membrane mimetic’ tools to stabilize membrane proteins in the absence of detergents by shielding their hydrophobic transmembrane regions from the aqueous environment [5,6,32]. The most established membrane mimetics are based on either proteins (nanodiscs and Salipro), short peptides (peptidiscs), or synthetic polymers (SMALPs). With different mimetics available, how do researchers decide which one is most suitable for their research question? Researchers routinely screen multiple options by ‘trial-and-error’ to determine which gives the best results [42,75].

Nanodiscs are the most established and widely used membrane mimetic, but protein reconstitution into nanodiscs can sometimes be challenging since many parameters must be optimized to ensure formation of homogenous particles. The Salipro and peptidisc systems offer more streamlined reconstitution procedures, with fewer parameters requiring optimization. However, a shortcoming shared by nanodiscs, Salipro, and peptidisc is that the membrane protein of interest must first be exposed to detergents prior to reconstitution. Despite its shortcomings, SMALPs (or ‘Lipodisqs’) remain the only membrane mimetic that can extract membrane proteins from cellular membranes in the absence of detergents.

## Perspectives

- *Importance of the field:* Membrane proteins are involved in many essential cellular processes and are important drug targets. However, they are more difficult to study than soluble proteins because they are embedded in hydrophobic cellular membranes. Membrane mimetics are useful tools for stabilizing membrane proteins outside their native environment in a water-soluble format that is compatible with downstream structural and biochemical analysis.
- *Summary of current thinking:* Several membrane mimetic systems have been developed in the last two decades. While these systems share some common properties, each has unique advantages and disadvantages. Researchers working in the membrane protein field should consider screening different membrane mimetics to determine which one works best for their membrane protein/complex of interest.
- *Future directions:* Membrane mimetics provide a robust platform for facilitating high-resolution structural characterization of membrane proteins and complexes, particularly by Cryo-EM. In the context of human health and disease, membrane mimetics may also be useful in the pharmaceutical industry for stabilizing medically relevant membrane proteins such as ion channels and GPCRs prior to screening for small molecule binders and/or antibody binders [68].

## Open Access

Open access for this article was enabled by the participation of University of Oxford in an all-inclusive *Read & Publish* agreement with Portland Press and the Biochemical Society under a transformative agreement with JISC.

## Abbreviations

CMC: critical micellar concentration
Cryo-EM: cryo-electron microscopy
MP: mass photometry
MSP: membrane scaffold protein
nMS: native mass spectrometry
NS-EM: negative stain electron microscopy
NSP: nanodisc scaffold peptide
NSP_r_: nanodisc scaffold peptide, reversed
SapNPs: Saposin lipid nanoparticles
SEC: size-exclusion chromatography
SMA: styrene maleic acid
SMALPs: SMA lipid particles
